# Skeletal muscle vulnerability in a child with Pitt-Hopkins syndrome

**DOI:** 10.1186/s13395-024-00348-0

**Published:** 2024-07-18

**Authors:** Celine Chiu, Alma Küchler, Christel Depienne, Corinna Preuße, Adela Della Marina, Andre Reis, Frank J. Kaiser, Kay Nolte, Andreas Hentschel, Ulrike Schara-Schmidt, Heike Kölbel, Andreas Roos

**Affiliations:** 1 Centre for Neuromuscular Disorders, Department of Pediatric Neurology, Centre for Translational Neuro- and Behavioral Sciences, University Hospital Essen, 45147 Essen, Germany; 2 Center for Rare Diseases Essen, Institute for Human Genetics, University Hospital Essen, University Duisburg-Essen, 45147 Essen, Germany; 3 Department of Neuropathology, Charité - Universitätsmedizin Berlin, corporate member of Freie Universität Berlin and Humboldt-Universität Zu Berlin, Charitéplatz 1, 10117 Berlin, Germany; 4 Department of Neuropediatrics, Charité-Universitätsmedizin Berlin, Corporate Member of Freie Universität Berlin, Humboldt-Universität Zu Berlin, Berlin Institute of Health (BIH), Augustenburger Platz 1, 13353 Berlin, Germany; 5 Institute for Human Genetics, University Hospital Erlangen, Friedrich-Alexander-University, 91054 Erlangen, Germany; 6 Department of Neuropathology, University Hospital Aachen, RWTH Aachen University, 52074 Aachen, Germany; 7 Leibniz-Institute for Analytical Science -ISAS- E.V, 44127 Dortmund, Germany; 8 Children’s Hospital of Eastern Ontario Research Institute, Ottawa, ON K1H 5B2 Canada; 9 Department of Neurology, Medical Faculty and University Hospital Düsseldorf, Heinrich Heine University, 40225 Düsseldorf, Germany

**Keywords:** Pitt Hopkins syndrome, TCF4, Muscle, Myogenesis, Muscle proteomics

## Abstract

**Background:**

TCF4 acts as a transcription factor that binds to the immunoglobulin enhancer Mu-E5/KE5 motif. Dominant variants in *TCF4* are associated with the manifestation of Pitt-Hopkins syndrome, a rare disease characterized by severe mental retardation, certain features of facial dysmorphism and, in many cases, with abnormalities in respiratory rhythm (episodes of paroxysmal tachypnea and hyperventilation, followed by apnea and cyanosis). Frequently, patients also develop epilepsy, microcephaly, and postnatal short stature. Although *TCF4* is expressed in skeletal muscle and TCF4 seems to play a role in myogenesis as demonstrated in mice, potential myopathological findings taking place upon the presence of dominant TCF4 variants are thus far not described in human skeletal muscle.

**Method:**

To address the pathological effect of a novel deletion affecting exons 15 and 16 of *TCF4* on skeletal muscle, histological and immunofluorescence studies were carried out on a quadriceps biopsy in addition to targeted transcript studies and global proteomic profiling.

**Results:**

We report on muscle biopsy findings from a Pitt-Hopkins patient with a novel heterozygous deletion spanning exon 15 and 16 presenting with neuromuscular symptoms. Microscopic characterization of the muscle biopsy revealed moderate fiber type I predominance, imbalance in the proportion of fibroblasts co-expressing Vimentin and CD90, and indicate activation of the complement cascade in *TCF4*-mutant muscle. Protein dysregulations were unraveled by proteomic profiling. Transcript studies confirmed a mitochondrial vulnerability in muscle and confirmed reduced *TCF4* expression.

**Conclusion:**

Our combined findings, for the first time, unveil myopathological changes as phenotypical association of Pitt-Hopkins syndrome and thus expand the current clinical knowledge of the disease as well as support data obtained on skeletal muscle of a mouse model.

**Supplementary Information:**

The online version contains supplementary material available at 10.1186/s13395-024-00348-0.

## Background

TCF4 (also known as E2-2, FECD3, ITF2, PTHS and SEF2 among others) is known to act as a transcription factor that binds to the immunoglobulin enhancer Mu-E5/KE5 motif. It activates gene transcription by binding to the Ephrussi-box (“E-box”, 5’-CANNTG-3’) and is itself widely expressed and involved in the initiation of neuronal differentiation [1, 2]. Already 30 years ago, an impact of TCF4 in muscle was demonstrated by linking TCF4 function to inhibition of MyoD, a muscle-specific transcription factor [3]. Along this line TCF4 expression in human muscle has already been demonstrated [4] and results of previous in vitro studies unravelled that the expression of different TCF4 isoforms is important for the differentiation process of C2C12 cells [5]. Furthermore, TCF4 expression has been described in mesodermal cell populations leading to limb muscle formation in vertebrates [6]. Genome-wide association studies have identified common *TCF4* variants as susceptibility loci for schizophrenia, Fuchs' endothelial corneal dystrophy, and primary sclerosing cholangitis [1]. However, defects in *TCF4* (NM_003199.2) are also causative for Pitt-Hopkins syndrome (PTHS, OMIM #610,954) [4, 7], a multisystem disorder characterized by severe mental retardation [8], and – in many cases – abnormalities in respiratory rhythm [9–11]. Patients also frequently develop epilepsy, ocular anomalies, postnatal macrosomia and present with distinctive facial features including a wide mouth and postnatal microcephaly [8, 9, 12]. Further neurological features include motor incoordination, seizures, typical behavior and subtle brain abnormalities [13]. The syndrome is caused by pathogenic heterozygous de novo variants in the *TCF4* gene (chromosome 18q21; NM_003199.2) encoding the transcription factor 4 and boys and girls appear to be affected equally [7].

Although, the role of TCF4 in muscle cell function and differentiation has been studied before (see above), investigations targeting the impact of TCF4 mutations on muscle cell integrity and potential myopathology in terms of potential skeletal muscle vulnerability in PTHS are still lacking in literature.

We report on the findings in a muscle biopsy derived from a boy with muscular hypotonia, apnoea, severe motor developmental delay and moderate response to pyridostigmine. This was indicative of the presence of a congenital myasthenic syndrome. The muscle biopsy was performed in the second year of life to rule out myopathy as the cause of the muscle weakness before his molecular genetic diagnosis was made. Microscopic investigation of this quadriceps muscle biopsy unveiled a predominance of type I fibers and complement activation. Transcript studies of *TCF4* demonstrated reduced level in muscle. Biochemical analysis of muscle protein extract via proteomics revealed dysregulation of different classes of proteins mainly hinting toward perturbed mitochondrial function, oxidative stress burden and complement activation. Immunofluorescence studies focusing on muscle fibroblasts revealed an imbalance of vimentin and CD90 co-immunoreactivity compared to controls. These combined clinical, microscopic, transcriptional, and biochemical findings indicate an affection of skeletal muscle upon the presence of a pathogenic *TCF4* variant (NM_003199.2) associated with the clinical manifestation of PTHS.

## Materials and methods

### Exome-sequencing

To confirm the clinical diagnosis of PTHS, we sequenced the exome of the patient. Library was prepared using the Twist Human Core Exome plus RefSeq Panel (Twist Bioscience, San Francisco, USA) and sequenced by paired-end 150 bp massively parallel sequencing on a NextSeq 2000 Instrument (lllumina). Reads were mapped on the hg19 reference genome and variants called using a commercially available (Varfeed) pipeline developed by Limbus Medical Technologies (Rostock, Germany). Variants were filtered using the Varvis software (Limbus Medical Technologies). This pipeline includes an analysis of copy number variants. Confirmation of the suspected copy number aberration in the index patient and parental analysis were performed by MLPA (kit P075, MRC Holland).

### Histology, enzyme histochemistry, immunofluorescence and electron microscopy

Histological studies and enzyme histochemistry and on the muscle biopsy were carried out according to standardized procedures. Immunofluorescence was carried out on 7 µm cryosections as described previously [14] making use of primary antibodies targeting C5b-9 (Dako, M0777), HLA1 (Dako, M0736), HLA2 (Dako, M0775), CD4 (Dako, M0716), CD8 (Novocastra/Leica, NCL-CD8-4B11), CD20 (Novocastra/Leica, NCL-CD20-L26), CD45 (Dako, M0701), CD68 (Dako, M0718), CD79A (Dako, M7050), and CD90 (Dianova, DIA-100), MYH7 (GeneTex, GTX100713) as well as Vimentin (GeneTex, GTX112661); secondary antibodies: Invitrogen, Alexa Fluor 488, A11029 and Invitrogen Alexa Fluor 488, A11008.

### Proteomic profiling

Protein extraction and subsequent mass spectrometry-based protein quantification studies were carried out as described previously [15]. For proteomic profiling, three control samples derived from male children of Caucasian origin (10 months, 12 months, and 14 months of age, respectively) with an unremarkable family history were included. In these control biopsies histological and laboratory investigations remained unremarkable. In brief, a bottom-up proteomic approach with label-free quantification of peptides was performed in a data-independent acquisition mode making use of an in-house generated peptide library.

For relative quantification, only proteins identified with two or more unique peptides were considered for further analysis. After this initial filtering, the average of the normalized abundances was calculated and used to determine the ratios between the overexpression model or patient samples with their respective controls including log2 transformation and p‐value generation by performing a Student's t‐test. For further proteomaps-based pathway analysis (www.proteomaps.net), we considered only proteins that were identified with two or more peptides in the muscle samples derived from the PTHS patient and controls.

### RNA extraction and quantitative reverse transcription PCR (qRT-PCR)

Total RNA was extracted from whole muscle specimens of the PTHS patient and juvenile control muscle (see above) using Trizol/chloroform extraction, followed by cDNA transcription using the High-Capacity cDNA Archive Kit (Applied Biosystems, Foster City, CA). For qPCR reactions, 10 ng cDNA was used for subsequent analysis on the Applied Biosystems™ QuantStudio™ 6 Flex Real-Time PCR System (ThermoFischer, Waltham, MA; USA, running conditions: 95 °C 0:20, 95 °C 0:01, 60 °C 0:20, 40 cycles). Targeted transcripts were run as triplicates and the reference gene *PGK1* has been included as internal control to normalize the relative expression of the targeted transcripts.

The TaqMan® Gene Exp Assay (Life Technologies/ThermoFisher) are as follows: *BNIP3/BCL2* Hs00198106_m1, *B2M* Hs00187842_m1, *C2* Hs00918862_m1, *C5* Hs01004342_m1, *CXCL12* Hs03676656_mH, *CXCR5* Hs00540548_s1, *ETFA* Hs00164511_m1, *LDHB* Hs00929956_m1, *MT-ND1* Hs02596873_s1, *NNMT* Hs00196287_m1, *NUDFA12* Hs07291325_m1, *PGK1* Hs99999906_m1, *TCF4* Hs00162613_m1. Displayed is the ratio (in %) of gene expression in skeletal muscle derived of the PTHS patient compared to the expression of the juvenile controls.

## Results

### Clinical findings

#### Development

The boy was born via cesarian section in 36th week of gestation due to HELLP-syndrome of the mother [weight 3060 g (25 P., -0.66 z), length 54 cm (85 P., -1.03 z), head circumference 35 cm (52 P., -0.04 z)] he suffered from episodes of apnea and bradycardia until 4 months of age and needed feeding via nasogastric tube for 6–8 weeks postnatally. Motor development was delayed, as he was able to sit with 22 months, to stand with 24 months and to walk with 30 months. Achievement of speech development milestones was also delayed: two words at the age of 15 months and no further development afterwards. In line with this, cognitive development is severely impaired (IQ 50/SON-R). Communication could be established using sign language. Neurologically, also at his last appointment at the age of 12 years in 2023, he presented with muscular hypotonia, hyperextensible joints and soft muscles. Subsequently, he had developed a broad-based gait pattern and difficulties in climbing stairs. The mother reported episodes suspicious of absence seizures with upward gaze deviation and tremor. EEG was also normal.

#### Diagnostic workup

**Fig. 1:**
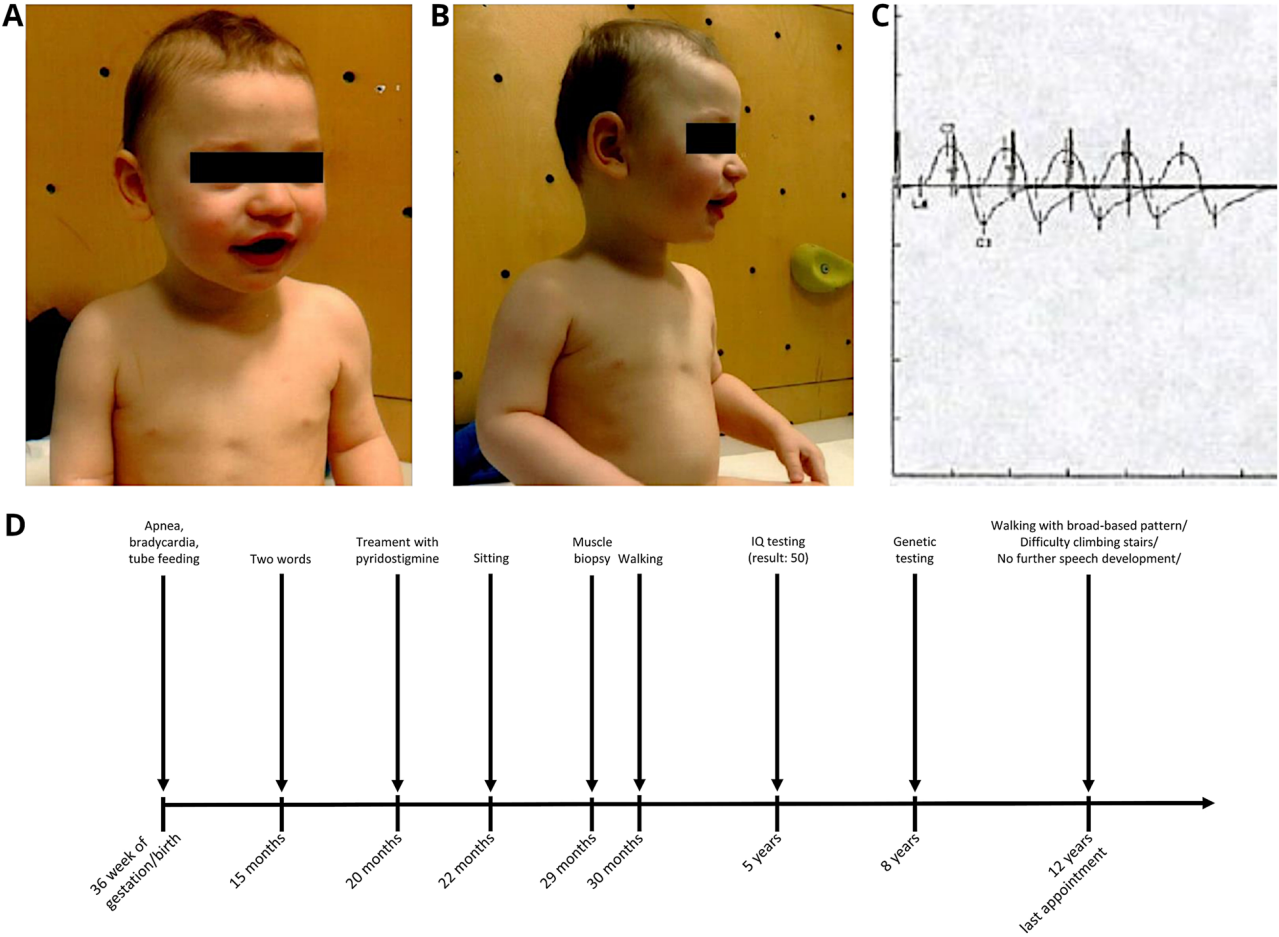
Patient’s photograph at the age of 15 months. **A** Facial dysmorphia with Cupid’s bow lip. **B** Doubled crease of M. pectoralis is shown. **C** Positive decrement of the median nerve upon repetitive stimulation. **D** Schematic representation of the diagnostic work-up along with clinical findings in our PTHS patient

At his first presentation in our department with the age of 15 months (Fig. 1A and B), the boy showed a severe motor development disorder with pronounced weakness in the upper extremities and generalized muscular hypotonia. A positive decrement (median nerve) was identified upon repetitive nerve stimulation (Fig. 1C). Based on this pathological decrement and his clinical features fitting to a congenital myasthenic syndrome, at the age of 20 months treatment with pyridostigmine (3.8 mg/ Kg bodyweight) was initiated and the boy initially (four weeks after first treatment) showed a benefit in muscle strength of lower extremities and improvement of motor development: he was able to drink on his own and roll from back to stomach as well as to sit unsupported and put himself into upward position. Repeatedly blood tests with creatine kinase (last CK-value: 161 U/l, ref < 165 U/l), basic and extended metabolic screening produced normal results. An MRI of the brain showed no pathologies (data not shown). EEG was normal. Electroneurography measuring nerve conduction velocity showed normal values.

However, as no persisting improvement was achieved upon the treatment with pyridinstigmine, a muscle biopsy was performed at the age of 2.5 years to exclude an underlying myopathic disease.

With disease progression, our follow-up clinical examinations revealed dysmorphia with persisting Cupid’s bow lip (Fig. 1), talipes varus of both feet and doubled crease of the pectoralis muscle. The patient was diagnosed with PTHS at the age of 8 years.

A schematic representation of the timeline of the diagnostic workup and clinical findings is presented in Fig. 1D.

### Microscopic findings

**Fig. 2:**
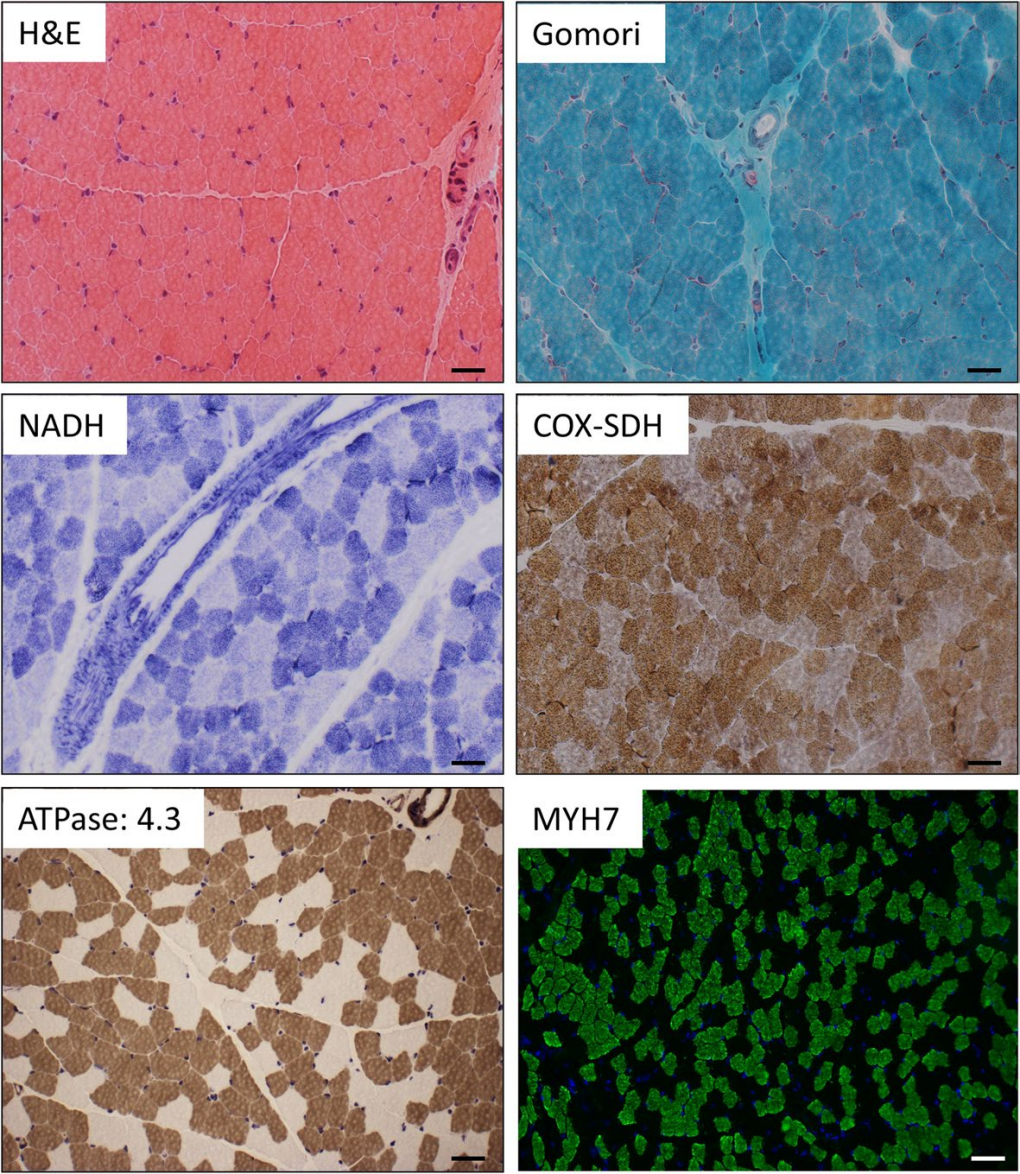
Light microscopic findings on *TCF4*-mutant muscle. H&E as well as Gomori stains show normal morphology. Increase of mitochondria in the subsarcolemmal region of some muscle fibres is indicated by NADH and COX-SDH stain. COX-negative fibers as not present. ATPase stain (pH 4.3) shows moderate increase of type I fibers occasionally organized in groups. This finding was confirmed by immunofluorescence studies of MYH7 labelling type I fibers. COX: cytochrome c oxidase; SDH: succinate dehydrogenase; HE: Hematoxylin and Eosin; Gomori: Gomori-Trichorme stain; NADH: Nicotinamid Adenin Dinucleotid Hydrid. Scale bars: 50 µm and in immunofluorescence stain of MYH7 100 µm

**Fig. 3:**
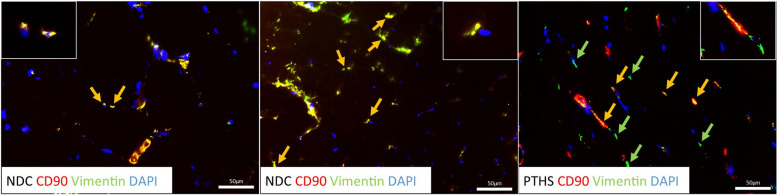
Immunofluorescence-based studies of myofibroblasts on *TCF4*-mutant muscle. Reduced co-localization of CD90 and Vimentin in the muscle biopsy specimen of the PTHS patient compared to sex- and age-matched normal disease controls (NDC). Nuclei are visualized by DAPI staining. Co-localization is indicated by yellow arrows whereas areas solely immunoreactive for Vimentin are highlighted with green arrows. Scale bars: 50 µm

Due to suspected muscular involvement, a muscle biopsy was performed at two years of age. Histological investigation of the muscle collected from right vastus lateralis muscle did not show any striking myopathologies. However, results of ATPase 4.3 reaction showed a moderate type I fiber predominance, whereby these fibers were occasionally organized in groups (Fig. 2). An enzyme histochemistry revealed no COX-negative fibers but indicated increased subsarcolemmal mitochondrial content in some fibers by nicotine adenine dinucleotide hydride (NADH) and combined Cytochrome C oxidase and Succinate dehydrogenase reaction (COX-SDH). Immunohistological-based examination of protein abundances (including Caveolin 3, Dystrophin 1–3, α-/ β-Dystroglycan, α-/ β-/ γ-/ δ- Sarcoglycan, Laminin α2, Laminin α5, Emerin, Dysferlin, Collagen IV and VI, Utrophin, neonatal myosin) of the muscle showed normal results (data not shown). Given that in the central nervous system an impact of TCF4 abundance on fibroblast growth factor production (necessary for neuronal function) was shown [16], by performing immunofluorescence studies on the patient-derived muscle biopsy, we additionally investigated the characteristics of muscle fibroblasts: Co-staining of Vimentin (fibroblast marker) and CD90 (marker of activated fibroblasts) revealed an approximate complete co-expression of both proteins in muscle biopsy specimen derived from two controls whereas in the biopsy derived from our patient a co-expression could only be detected in roughly 50% of fibroblasts with a higher proportion solely showing immunoreactivity for CD90 but not Vimentin (Fig. 3).

### Molecular genetic findings

Conventional karyotyping and array CGH were unremarkable. The boy was tested negative for Angelman syndrome and panel diagnostic for encephalopathic epilepsies including Rett syndrome and Coffin-Lowry syndrome. The analysis of point variants in genes relevant for PTHS did not reveal any likely pathogenic or pathogenic variants. However, the analysis of copy number variants revealed a possible deletion of exons 15 and 16 of TCF4 [(NM_003199.2): c.(1146 + 1_1147-1)_(1468 + 1_1487-1)del]. This deletion was confirmed by MLPA analysis and shown to be absent in both parents.

### Proteomic signature of *TCF4*-mutant muscle

**Fig. 4:**
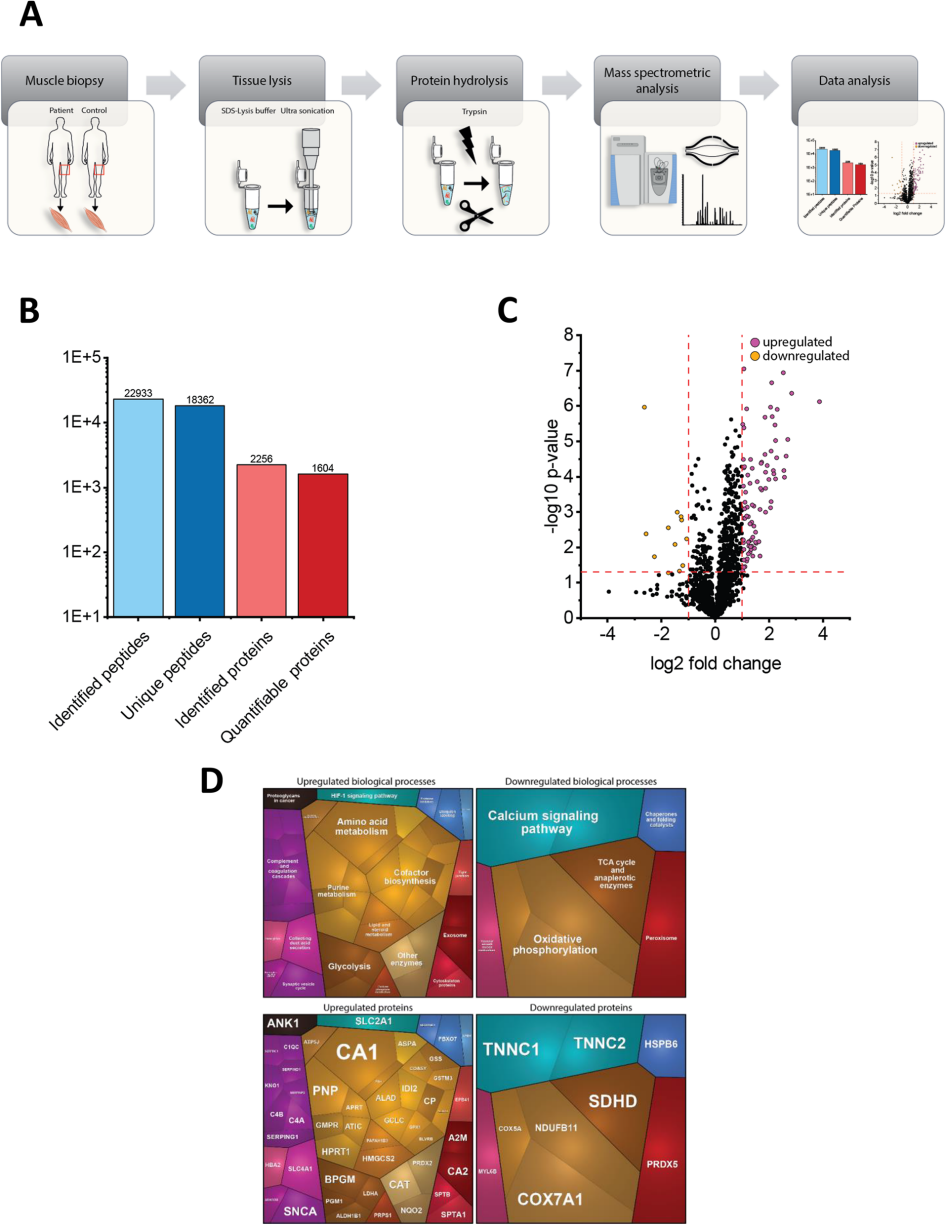
Proteomic studies on whole protein extract of *TCF4*-mutant muscle. **A** Schematic representation of the applied workflow. **B** Overall statistics of PTHS skeletal muscle proteomics. **C** Volcano plot showing proteins identified as being decreased (orange dots) and increased (purple dots) in PTHS patient-derived muscle. **D** Results of proteomaps-based data analysis indicating activation of complement cascade and altered oxidative phosphorylation based on identified protein dysregulations

A proteomic profile was performed on whole protein extract of the muscle biopsy of the PTHS patient and three controls and enabled the robust quantification of 2243 proteins (Fig. 4A and B). Among these, we identified 99 statistically significant dysregulated proteins: 87 Proteins were upregulated, 12 proteins downregulated (Fig. 4C & Supplementary Table 1). Of these dysregulated proteins, 17 are involved in B-cell-depending immune response, including immunoglobulins, but also unspecific humoral immune system, such as complement factors (Table 1 & Fig. 4D). Notably, 7 dysregulated proteins modulate oxidative stress burden (Table 2) and further 7 affected proteins are localized to mitochondria (Table 3). After filtering the proteomic data for significantly dysregulated proteins with known involvement in specific diseases, we identified different proteins associated with phenotypical features of PTHS: These proteins include NAXE (NADPH-hydrate epimerase) and HMGCS2 (Hydroxymethylglutaryl-CoA synthase). Pathogenic *NAXE* variants are associated with PEBEL (encephalopathy, progressive, early-onset, with brain edema and/or leukoencephalopathy) (OMIM #617,186) [17]. Mitochondrial HMG-CoA synthase deficiency caused by bi-allelic pathogenic variants may be associated with psychomotoric retardation (OMIM # 605,911). GSS (Glutathione synthetase) is related to glutathione synthetase deficiency, which go along with central nervous damage [18]. Psychomotoric retardation and muscle weakness are associated with CAH2 (carbonic anhydrase II)-deficiency [19]. Remarkably some of the upregulated proteins are in line with inflammatory processes. In particular, 15 immunoglobulin subtypes were found to be increased in the context of possible B-cell activation (Table 1). Two complement factors (C4A and C4B) were found to be upregulated according to humoral immune response. None of those dysregulated proteins is notoriously associated with neuromuscular diseases on a genetic base, but B cell and complement activation are well known hallmarks in a variety of muscular diseases.

**Table 1:** Dysregulated proteins within PTHS patient-derived muscle biopsy associated with inflammatory processes

UniProt Entry	Protein Accession Number	OMIM#	Gene Name	Patient vs. Control	*p*-Value	Dysregulation status
IGHA1	P01876	146,900	IGHA1	7.26	0.000	upregulated
IGHM	P01871	147,020	IGHM	6.29	0.000	upregulated
IGHA2	P01877	147,000	IGHA2	5.85	0.000	upregulated
IGLL5	B9A064	NA	IGLL5	4.78	0.000	upregulated
IGHG4	P01861	147,130	IGHG4	4.59	0.000	upregulated
IGHG1	P01857	147,100	IGHG1	4.31	0.000	upregulated
IGHG2	P01859	147,110	IGHG2	4.26	0.000	Upregulated
LV147	P01700	NA	IGLV1-47	4.20	0.000	upregulated
KV320	P01619	NA	IGKV3-20	3.87	0.000	upregulated
IGLC2; IGLC3	P0DOY2; P0DOY3	NA	IGLC2; IGLC3	3.66	0.000	upregulated
HV551	A0A0C4DH38	NA	IGHV5-51	3.24	0.000	upregulated
CO4B	P0C0L5	120,820	C4B	3.18	0.018	upregulated
HV366; HV353; HV333; HVC33	A0A0C4DH42; P01767; P01772; P0DP02	NA	IGHV3-66; IGHV3-53; IGHV3-33; IGHV3-30–3	2.76	0.011	upregulated
IGKC	P01834	NA	IUGKC	2,63	0.000	upregulated
CO4A	P0C0L4	120,810	C4A	2,53	0.006	upregulated
HV315	A0A0B4J1V0	NA	IGHV3-15	2,37	0.007	upregulated
HV434	P06331	NA	IGHV4-34	2,09	0.023	upregulated

*N/A* Not applicable

**Table 2:** Dysregulated proteins within PTHS patient-derived muscle biopsy associated with oxidative stress

UniProt Entry	Protein Accession Number	OMIM#	Gene Name	Patient vs. Control	*p*-Value	Dysregulation status
CAH1	P00915	114,800	CA1	15.82	0.000	upregulated
CAH2	P00918	611,492	CA2	4.88	0.000	upregulated
PRDX2	P32119	600,538	PRDX2	4.36	0.000	upregulated
CATA	P04040	115,500	CAT	4.04	0.000	upregulated
GPX1	P07203	138,320	GPX1	2.21	0.000	upregulated
GSHB	P48637	601,002	GSS	2.16	0.001	upregulated
GSTM3	P21266	138,390	GSTM3	2.09	0.001	upregulated

**Table 3:** Dysregulated proteins within PTHS patient-derived muscle biopsy showing mitochondrial localization

UniProt Entry	Protein Accession Number	OMIM#	Gene Name	Patient vs. Control	*p*-Value	Dysregulation status
HMCS2	P54868	600,234	HMGCS2	4.67	0.000	Upregulated
NNRE	Q8NCW5	608,862	NAXE	2.00	0.006	Upregulated
NDUBB	Q9NX14	300,403	NDUFB11	0.48	0.006	Downregulated
CX7A1	P24310	123,995	COX7A1	0.44	0.032	Downregulated
PRDX5	P30044	606,583	PRDX5	0.42	0.002	Downregulated
DHSD	O14521	602,690	SDHD	0.38	0.001	Downregulated
COX5A	P20674	603,773	COX5A	0.16	0.000	Downregulated

### Results of confirmational immunofluorescence studies on *TCF4*-mutant muscle

**Fig. 5:**
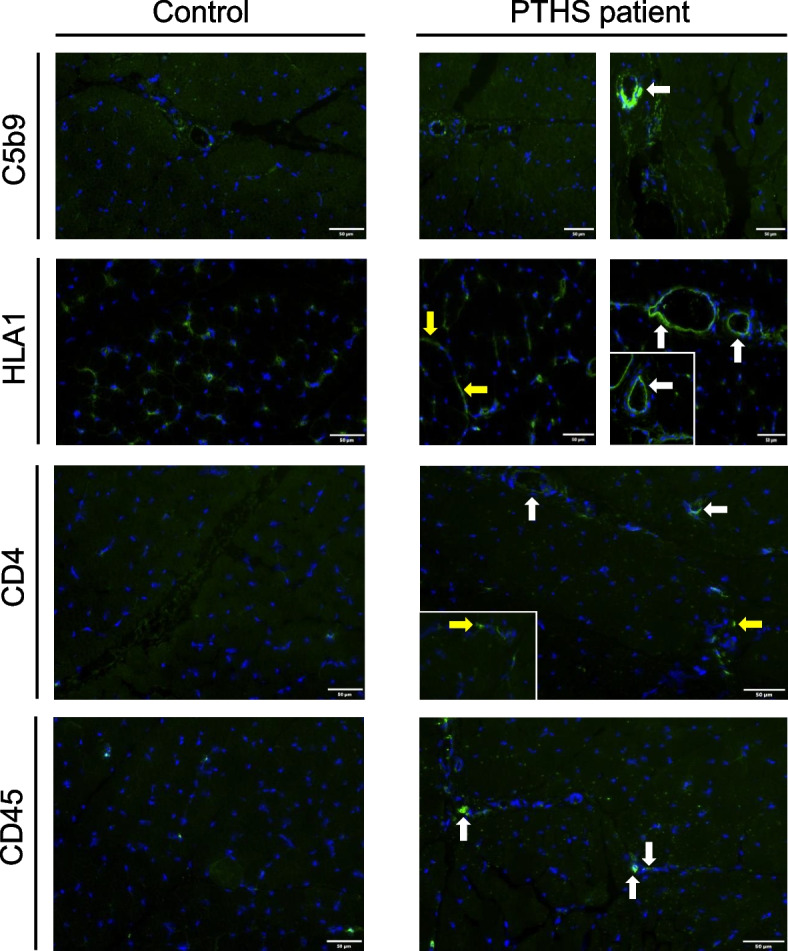
Immunofluorescence studies on *TCF4*-mutant muscle. C5b-9 immunostaining shows an increased immunoreactivity at some vessels (white arrow). Similarly, HLA1 and CD4 showed an increase at vessels (white arrows) accompanied by a general increase within the extracellular space (yellow arrows) which are indicative for the presence of CD4 + helper T cells. Immunostaining of CD45 revealed a dot-like increased immunoreactivity within the extracellular space (white arrows). Scale bars: 50 µm

Given that our proteomic findings were indicative for activation of the complement cascade and B-cell involvement, further immunostaining studies were carried out to validate these findings in the muscle tissue. C5b-9 immunostaining studies revealed an increased expression on some larger vessels, but not on muscle fibers (Fig. 5). Also, HLA1 showed more intense expression on larger vessels (Fig. 5, white arrows) as well as an increased intensity on smaller capillaries (Fig. 5, yellow arrows). Only singular CD4 + T-cells were detected; however, the few detected cells were localized around the vessels (Fig. 5). Immunostaining for the lymphocyte marker CD45 only reveals single immunoreactive cells in the muscle tissue (Fig. 5), while immunoreactivity for the B-cell marker CD20 was not detected (data not shown). Immunostaining of HLA2 was unremarkable and staining for CD8 + T cells, as well as CD68 + macrophages and CD79A + plasma cells was negative (data not shown).

**Fig. 6:**
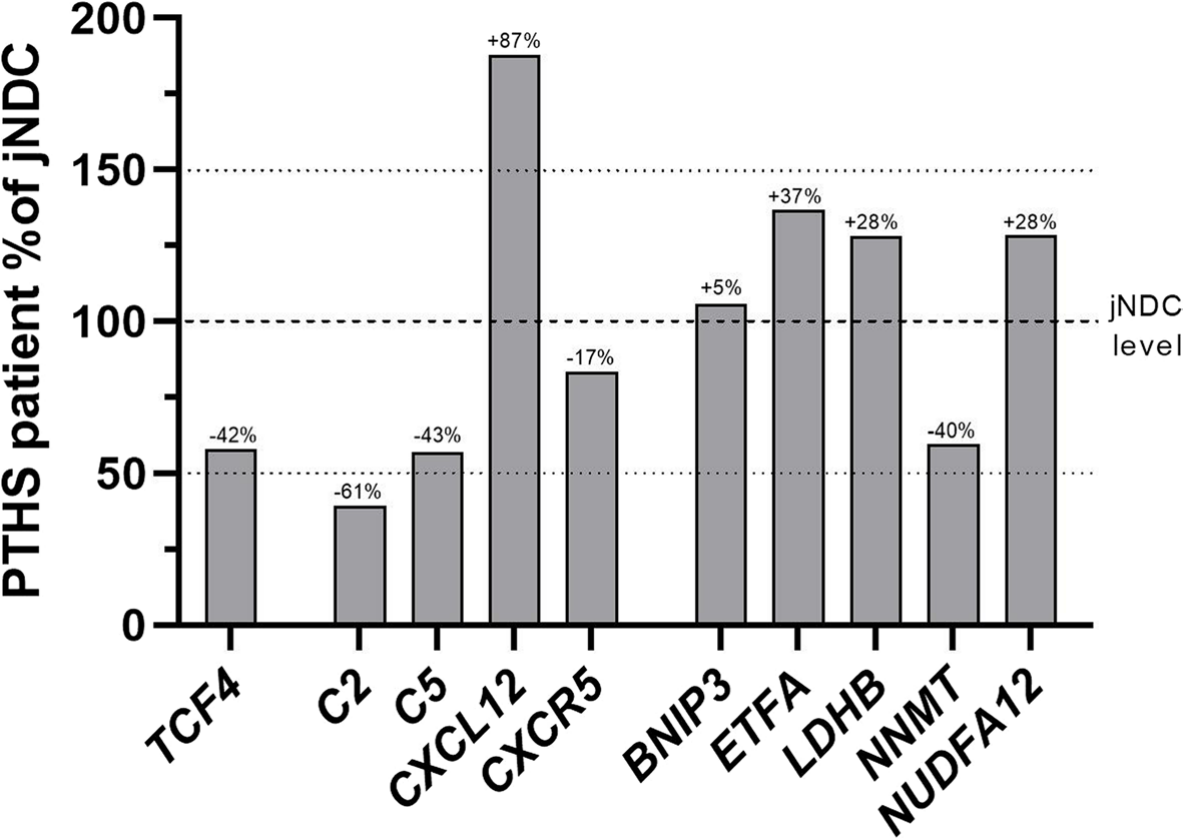
Transcript studies on *TCF4*-mutant muscle. Quantitative PCR studies on whole RNA-extracts converted to cDNA revealed a 42% reduction of *TCF4* transcripts along with a reduction of *C2* (-61%), *C5* (-43%), *CXCR5* (-17%) and *NNMT* (-40%). In contrast, level of further studied transcripts were increased: *CXCL12* (+ 87%), *ETFA* (+ 37%), *LDHB* (+ 28%) and *NDUFA12* (+ 28%). *BNIP3* showed only a 5% increase

To study the effect of the exon deletion in *TCF4* on the level of corresponding transcripts, quantitative PCR was carried our revealing a profound reduction of the gene expression levels, compared to the level detected in muscle of juvenile control individuals (Fig. 6).

To validate proteomic findings, we additionally studied the level of transcripts corresponding to components of the complement cascade (*C2*, *C5*), B cell homing factor *CXCL12* and it´s receptor *CXCR5*, as well as genes responsible for mitochondrial function and turnover (*ETFA*, *NNMT*, *NDUFA12*, *BNIP3* and *LDHB*). Whereas level of *C2* and *C5* were profoundly decreased (61% and 43%, respectively), the chemokine *CXCL12* was strongly increased, hinting to a recruitment of B cells into the muscle tissue. However, the respective receptor *CXCR5* was only slight changed (-17%) compared to the level identified in control muscle (Fig. 6). Regarding the level of transcripts encoding for mitochondrial relevant proteins, only *NNMT* displayed a decreased (by 40%) whereas *ETFA*, *NDUFA12* and *LDHB* showed an increased (37%, 28% and 28%, respectively) (Fig. 6). Level of *BNIP3* remained almost stable compared to controls (Fig. 6).

## Discussion

In the present study, we introduce a new case of PTHS based on a heterozygous intragenic deletion affecting exons 15 and 16 of the *TCF4* gene. This variant occurred de novo and is in line with the overall clinical presentation of our patient thus expanding the current genetic landscape of *TCF4*-associated PTHS. Studies of *TCF4* transcript level revealed a 42% decrease compared to the level detected in muscle samples derived from age-matched control individuals. This molecular finding suggests that a profound decrease of the amount of *TCF4* transcript level constitutes the molecular cause of the manifestation of the disease in our patient and is in line with previous descriptions (OMIM #610,954). The clinical presentation of our case included delayed motor development, muscle hypotonia and muscular weakness. Prompted by the known role of TCF4 in muscle cell differentiation [5] (and the evidence that cancer cachexia is promoted by TCF4 which modulates expression of muscle atrophy promoting hormones [20], we – in the light of muscle weakness present in our patient – elucidated skeletal muscle vulnerability in PTHS by making use of a quadriceps biopsy. This biopsy was collected in the diagnostic work-up prior the molecular genetic diagnosis was made. Hence, we performed the most detailed examination of human skeletal muscle existing for *TCF4* pathology thus far. Our microscopic studies reveled increase of type I fibers (occasionally in appearing in groups).

Skeletal muscle comprises a heterogeneous population of myoblasts and fibroblasts and whereby fibroblasts have shown to role play a role maintaining cytokine balance and consequently promoting angiogenesis in the skeletal muscle [21]. This in addition to the known impact of TCF4 abundance on fibroblast growth factor production (necessary for neuronal function) was shown [16] prompted us to investigate myofibroblasts in the muscle biopsy of our PTHS patient more detailed: co-staining of CD90 and Vimentin revealed a reduced co-immunoreactivity accompanied by the increase of areas which are solely reactive for CD90 compared to investigated controls. As Vimentin plays a crucial role in fibroblast ageing and cell migration [22], one might speculate that this microscopic finding hints toward a fibroblast-modulated maturation defect of *TCF4*-mutant musculature. Our PTHS patient presents with a predominance of slow-twitch type I fibers and reduced *TCF4* level as highlighted by our transcript studies. Thus, our findings indicate an interplay of reduced TCF4 level, muscle fibre predominance and altered biochemical properties of myofibroblasts.

Based on our proteomic data, proteins indicative for B-cell activation and inflammation such as immunoglobulins are increased accompanied by factors belonging to the complement cascade. Our immunostaining-based confirmational studies confirmed activation of complement cascade at the larger vessels (by staining of C5b-9). Vessels also showed increased immunoreactivity of HLA1, and we moreover identified an increase of areas immunoreactive for CD90, a membrane glycoprotein which is not only expressed in fibroblasts but also in activated endothelial cells [23]. These findings accord with altered biochemical characteristics of myofibroblast in *TCF4*-mutant muscle as demonstrated by the results of our immunofluorescence studies and moreover suggest that this may impact on vessel integrity which is also altered in our patient. Of note the cytokine/ chemokine *CXCL12*, which is essential for an inflammatory process by cell recruitment and homing of B-cells was strongly upregulated in the patient; however, histological staining of CD20 + B-cells did not reveal presence of these cells around the vessels while only singular CD4 + T-cells were detected around the vessels.

Immunostaining for the lymphocyte marker CD45 reveals the presence of only single immunoreactive cells within the muscle tissue. Most likely, these cells participate in the immune response and react to the complement activation. Taken together, although TCF4 is known to play a role in the negative regulation of proinflammatory cytokines [24], a strong and detrimental inflammation was not visible, since we did not detect any macrophages or intense infiltration of T-cells in the muscle biopsy of our patient.

Furthermore, mild mitochondrial affection could be found, which displayed as a combined histological, proteinogenic and transcriptional (increase of the metabolomic relevant factors *ETFA*, *NDUFA12* and *LDHB*) finding (Figs. 2, 3 and 6 as well as Table 3). However, this finding is rather non-specific but may accord with the observed fiber type I predominance upon presence of the heterozygous *TCF4* variant as these fibers notoriously display a high oxidative/ mitochondrial activity. This assumption is also supported by decreased level of *NNMT* encoding for Nicotinamide N-methyltransferase which shunts the NAD^+^ precursor NAM away from NAD^+^ biosynthesis through methylation and the knowledge that limited NAD^+^ affects in vivo energetics and mitochondrial function in *mdx* mice [25]. Stable *BNIP3* level suggest that altered mitophagy is rather unlikely in the mitochondrial affection of *TCF4*-mutant skeletal muscle. Indeed, results of our proteomic profiling approach also confirmed a dysregulation of mitochondrial proteins presumably impacting on metabolic activity and oxidative balance in *TCF4*-mutant muscle (Table 2 and Supplementary Table 1). Given that a dysregulation of mitochondrial proteins often indicates increased oxidative stress burden, upregulation of proteins dealing with degradation of toxic oxidative metabolites (Table 2) may hint toward altered mitochondria proteostasis concomitant with increased oxidative stress burden in *TCF4*-related muscle cell vulnerability. However, further functional studies – for instance on an established animal model [26] – are crucial to draw final conclusions.

## Conclusions

In conclusion, by introducing a novel intragenic variant (de novo deletion von exons 15 to 16) leading to haploinsufficiency and associated with the clinical manifestation of PTHS, we expand the current molecular genetic landscape of *TCF4* (NM_003199.2). Clinical, microscopic, transcriptional, and biochemical work-up of our case indicated an affection of skeletal musculature mainly prompted by fiber type I predominance accompanied by increased mitochondrial content identified on the transcript, the microscopic and the protein level. Type I fiber predominance accords with the results of previous in vivo studies on reduced Tcf4 level impacting on expression of slow myosin heavy chain. In addition, our combined proteomic, transcriptional and immunofluorescence findings indicate activation of the complement cascade in *TCF4*-mutant muscle mainly characterized by C5b-9 and HLA1 immunoreactive deposits at vessels. Nevertheless, it remains unclear, how far the dimensions of the dysregulated proteins affect the clinical phenotype, which requires further investigation. More studies focusing on potential myopathology in additional PTHS patients (or a suitable animal model) are crucial to delineate the impact of skeletal muscle involvement in the phenotypic spectrum of PTHS.

### Supplementary Information

Supplementary Material 1: Supplementary Table 1: List of overall proteomic findings obtained on *TCF4*-mutant muscle.

## Data Availability

No datasets were generated or analysed during the current study.

## Abbreviations

CGH: Comparative Genomic Hybridization
ETFA: Electron-transfer-flavoprotein, alpha subunit
HELPP-syndrome: Hemolysis, Elevated Liver enzymes and Low Platelets) syndrome
HMG-CoA: 3-hydroxy-3-methylglutaryl coenzyme A
LDHB: Lactate Dehydrogenase B
NDUFA12: NADH:Ubiquinone Oxidoreductase Subunit A12
MLPA: multiplex ligation-dependent probe amplification
PTHS: Pitt Hopkins Syndrome
P: Percentile
PCR: polymerase chain reaction
qRT-PCR: quantitative reverse transcription PCR
SON-R: Snijders-Oomen Non-verbal intelligence test
TCF4: Transcription factor 4
